# Biocompatibility, bioactivity and immunomodulatory properties of three calcium silicate-based sealers: an in vitro study on hPDLSCs

**DOI:** 10.1007/s00784-024-05812-1

**Published:** 2024-07-06

**Authors:** Alejandro Mora, David García-Bernal, Francisco Javier Rodríguez-Lozano, José Luis Sanz, Leopoldo Forner, James Ghilotti, Adrián Lozano, Sergio López-García

**Affiliations:** 1 Department of Dermatology, Stomatology, Radiology and Physical Medicine, Morales Meseguer Hospital, Faculty of Medicine, University of Murcia, Murcia, 30008 Spain; 2 Department of Biochemistry, Molecular Biology B and Immunology, Faculty of Medicine, Biomedical Research Institute (IMIB), University of Murcia, Murcia, 30120 Spain; 3 Department of Stomatology, Faculty of Medicine and Dentistry Universitat de València, C/ Gascó Oliag 1, Valencia, 46010 Spain

**Keywords:** Ceraseal, Totalfill BC sealer, WellRoot ST, Bioacompatibility, Bioactivity, Anti-inflammatory

## Abstract

**Objectives:**

To assess the biocompatibility, bioactivity, and immunomodulatory properties of three new calcium silicate cement-based sealers: Ceraseal (CS), Totalfill BC Sealer (TFbc) and WellRoot ST (WR-ST) on human periodontal ligament stem cells (hPDLSCs).

**Materials and methods:**

HPDLSCs were isolated from extracted third molars from healthy patients. Eluates (1:1, 1:2, and 1:4 ratio) and sample discs of CS, TFbc and WR-ST after setting were prepared. A series of assays were performed: cell characterization, cell metabolic activity (MTT assay) cell attachment and morphology (SEM assay), cell migration (wound-healing assay), cytoskeleton organization (phaloidin-based assay); IL-6 and IL-8 release (ELISA); differentiation marker expression (RT-qPCR assay), and cell mineralization (Alizarin Red S staining). HPDLSCs cultured in unconditioned (negative control) or osteogenic (positive control) culture media were used as a comparison. Statistical significance was established at *p* < 0.05.

**Results:**

All the tested sealers exhibited similar results in the cytocompatibility assays (cell metabolic activity, migration, attachment, morphology, and cytoskeleton organization) compared with a negative control group. CS and TFbc exhibited an upregulation of at least one osteo/cementogenic marker compared to the negative and positive control groups. CS and TFbc also showed a significantly higher calcified nodule formation than the negative and positive control groups. Both the marker expression and calcified nodule formation were significantly higher in CS-treated cells than TFbc treated cells. WR-ST exhibited similar results to the control group. CS and TFbc-treated cells exhibited a significant downregulation of IL-6 after 72 h of culture compared to the negative control group (*p* < 0.05).

**Conclusion:**

All the tested sealers exhibited an adequate cytocompatibility. CS significantly enhances cell differentiation by upregulating the expression of key genes associated with bone and cementum formation. Additionally, CS was observed to facilitate the mineralization of the extracellular matrix effectively. In contrast, the effects of TFbc and WR-ST on these processes were less pronounced compared to CS. Furthermore, both CS and TFbc exhibited an anti-inflammatory potential, contributing to their potential therapeutic benefits in regenerative endodontics.

**Clinical relevance:**

This is the first study to compare the biological properties and immunomodulatory potential of Ceraseal, Totalfill BC Sealer, and WellRoot ST. The results act as supporting evidence for their use in root canal treatment.

## Introduction

Human periodontal ligament stem cells (hPDLSCs) represent a cost-effective source of stem cells that can be extracted and cultivated in vitro. These cells exhibit a high proliferative ability, along with a diverse differentiation potential, playing a critical role in the healing process of apical periodontitis [1]. The application of root filling materials can induce hPDLSCs to differentiate into osteoblasts and cementoblasts, thereby isolating the root canal from adjacent tissues and facilitating the repair of damaged apical tissues [2].

Calcium silicate-based sealers, also known as bioceramics, have achieved notable success in clinical endodontic applications [3]. Diverse formulations of bioceramics, including both powder-liquid and premixed variants, have been introduced to the field [4]. These materials, characterized by their high content of calcium di- and trisilicates, hydroxyapatite, alumina, glass ceramics, zirconia, bioactive glass, and calcium phosphates, have been engineered to facilitate tissue repair via the deposition of mineralized tissue [5, 6]. Not only do these substances precipitate into calcium and phosphate, thereby promoting the remineralization of hard tissues, but they also exhibit immunomodulatory properties [7]. Consequently, the therapeutic efficacy of bioceramic sealers has been ascribed to their anti-inflammatory and modulatory characteristics, which are integral to maintaining bone homeostasis and enhancing the regeneration of impaired tissues [7].

In vitro experiments are a reliable technique for evaluating various characteristics of novel materials [8]. The International Organization for Standardization (ISO) has established specific protocols to ensure consistency and replicability in the laboratory testing of biomaterials [9]. These protocols categorize cellular interactions into three types: extract-based, direct contact, and indirect contact. Extract-based methods expose cells to varying concentrations of a material, mimicking the diffusion of substances through tissues, particularly in scenarios involving irrigated tissues [10]. Assessing cell viability is crucial in the selection process for new materials and provides vital preliminary information before proceeding to clinical trials. Furthermore, there exists a diverse array of assays to measure cell viability, each relying on different aspects of cellular functionality [11–13].

Accordingly, the objectives of the present study were to evaluate the biological and immunomodulatory characteristics of three bioceramic sealers. The null hypotheses were that the materials and dilutions did not present significant differences in terms of biological and/or immunomodulatory properties.

## Materials and methods

### Cell culture and characterizations of hPDLSCs

The protocol for isolating human periodontal ligament stem cells (hPDLSCs) was approved by the University of Murcia Ethics Committee (IRB number: 3686/2021). Each participant provided written consent prior to their involvement in the research. hPDLSCs were harvested from the periodontal tissues of impacted wisdom teeth in a group of patients (*n* = 10; aged 16–22 years) and were cultivated in DMEM (Sigma-Aldrich Corporation, St. Louis, MO, USA), enriched with 10% fetal bovine serum (Sigma-Aldrich Corporation, St. Louis, MO, USA) and 1% penicillin/streptomycin (Gibco-BRL, Gaithersburg, MD, USA). In subsequent steps, 1 × 10^5^ cells were suspended in 100 mL of phosphate buffered saline (PBS; Sigma-Aldrich Corporation, St. Louis, MO, USA) with 1% fetal bovine serum (FBS; Sigma-Aldrich Corporation, St. Louis, MO, USA)). A mix of specific monoclonal antibodies linked to fluorescent markers (CD14, CD20, CD34, CD45, CD73, CD90, and CD105; Miltenyi Biotec, Bergish Gladbach, Germany) was then applied to affirm the mesenchymal characteristics of hPDLSCs using flow cytometry as previously reported [3].

### Material extracts and preparation

The tested endodontic sealers were Ceraseal (CS), TotalFill BC Sealer (TFbc) and Well-Root ST (WR-ST). Data regarding their manufacturers, compositions and batch numbers are shown in Table 1. Thirty cylindrical molds made of medical-grade rubber, each with dimensions of 5 mm in diameter and 2 mm in height, were fabricated using sterile syringe tubes as a reference for the dimensions. These molds underwent a sterilization process involving ultraviolet light exposure for a duration of 30 min. Following the manufacturers’ guidelines, sealers were mixed and allowed to set. Subsequently, each formed disc was individually placed in a distinct well within a 24-well plate, followed by submersion in a newly prepared growth medium at a temperature of 37 °C for a period of 24 h. Following ISO 10993-12 guidelines, a ratio of contact area of the discs/liquid (medium) of 1.25 cm^2^/ml was used. The initial extracts, with a ratio of 1:1, were obtained in compliance with ISO 10993-5 standards. Thereafter, various concentrations of these extraction media, specifically 25%, 50%, and 100% volume/volume, were generated as previously reported [14].

**Table 1:** Tested materials

Materials	Manufacturer	Composition	Lot Number
Ceraseal	Meta Biomed Co., 270, Osongsaengmyeong 1-ro, Osong-eup, Heungdeok-gu, Cheongju-si, Chungcheongbuk-do, South Korea	Calcium silicates, zirconium oxide, thickening agent	CSL2301077
TotalFill BC Sealer	Innovative BioCeramix Inc. 101–8218 North Fraser Way Burnaby, BC V3N 0E9 Canada	Zirconium oxide, Tricalcium silicate, Dicalcium silicate, Calcium hydroxide.	22003SP
Well-Root ST	Vericom CO., LTD. 48, Toegyegongdan 1-gil, Chucheon-Si, Gangwon-Do, Korea.	Calcium aluminosilicate compound, zirconium oxide, Filler and thickening agent.	WR316100

### Cytocompatibility test

The evaluation of the metabolic activity of human periodontal ligament stem cells (hPDLSCs) in presence of bioceramic sealers was performed utilizing an MTT assay (M5655-1G; Sigma-Aldrich, St. Louis, MO, United States). In summary, hPDLSCs were distributed in 96-well culture plates (Sigma-Aldrich, St. Louis, MO, United States), with each well containing 5 × 10³ cells. These cells were then subjected to varying concentrations (25%, 50%, and 100%) of each sealer extract over time intervals of 24, 48, and 72 h. As a control, cells maintained in complete medium without exposure to any of the sealers were employed. At predetermined timepoints, a concentration of 5 mg/mL of MTT reagent was introduced to each well. Subsequently, the plates were incubated at a temperature of 37 °C and 5% CO₂ atmosphere for 4 h. Lastly, the assessment of mitochondrial activity was carried out using a microplate reader (ELx800, Bio-Tek Instruments, Winooski, VT, United States) at a wavelength of 570 nm.

### Cell migration

The ability of human periodontal ligament stem cells (hPDLSCs) to migrate in the presence of bioceramic sealers was assessed through in vitro wound healing assays. The hPDLSCs were allocated into 12-well plates, with each well containing 2 × 10⁴ cells. An artificial wound was created in each well using a sterile pipette tip of 100 µL capacity. The progress of wound closure in both the experimental and control groups was documented at intervals of 0, 24, 48, and 72 h. These images were then analyzed quantitatively using the ImageJ software, developed by the National Institutes of Health, Bethesda, MD, United States. This procedure was replicated three times for each material and experimental condition.

### F-actin cytoskeleton staining

Staining of F-actin in the cytoskeleton was evaluated using a phalloidin-based assay to observe changes in the cytoskeleton of hPDLSCs following their exposure to pure (1:1) bioceramic material, as described in previous studies [15]. Initially, the cells underwent treatment for 72 h, after which they were fixed using 4% paraformaldehyde (PFA) (Merck Millipore, Darmstadt, Germany), for a 10-minute period. Following fixation, a 30-minute blocking stage was implemented using 5% bovine serum albumin (BSA) (Sigma-Aldrich, St. Louis, MO, United States). The cells were then incubated with TRITC-conjugated phalloidin (Invitrogen, Carlsbad, CA, United States), for one hour, while the control group was treated with phosphate-buffered saline. Nuclear staining was subsequently performed using 4,6-diamidino-2-phenylindole dihydrochloride (DAPI) (ThermoFisher, Waltham, MA, USA). The final stage involved capturing fluorescent images of the stained cells using advanced confocal microscopy (Leica, Wetzlar, Germany).

### Scanning electronic microscopy

hPDLSCs were seeded at a density of 5 × 10^3^ cells per well onto sealer disc surfaces (*n* = 9) within 48-well plates. Subsequently, the plates were placed in a 5% CO_2_ incubator at 37 °C for a period of 72 h to facilitate cell attachment. Post-incubation, the sealer discs bearing the hPDLSCs underwent a triple wash using phosphate-buffered saline (PBS). The cells were then fixed using a 2.5% glutaraldehyde solution in 0.1 M sodium cacodylate buffer. Following fixation, the samples were subjected to a dehydration process using ethanol and subsequently underwent hexamethyldisilane-assisted drying. The fully dried samples were sputter-coated with a gold and palladium mixture for enhanced conductivity. Finally, the samples were systematically evaluated using a scanning electron microscope (Jeol 6100 EDAX; Jeol Inc.). This analysis was conducted across three distinct regions of each sample, employing magnification levels of 100×, 300×, and 1500× to ensure comprehensive observation.

### Elisa assays

To evaluate the ability of human periodontal ligament stem cells (hPDLSCs) to secrete anti-inflammatory molecules, an Enzyme-Linked Immunosorbent Assay (ELISA) was employed. The supernatants for this assay were procured following the incubation of hPDLSCs in complete medium for a duration of 72 h at 37 °C, under identical conditions as previously described. The concentrations of the proinflammatory cytokines Interleukin 6 (IL-6) and Interleukin 8 (IL-8) present in these supernatants were quantified utilizing specific human ELISA kits (Elabscience, Bethesda, MD, United States). The absorbance readings, indicative of cytokine levels, were obtained at a wavelength of 450 nm using a microplate reader. This methodology facilitated a precise quantification of IL-6 and IL-8, thus assessing the anti-inflammatory secretion profile of the hPDLSCs.

### Gene expression profiling

At the conclusion of 3 and 14 days of incubation with the designated experimental sealers (*n* = 3), total RNA was extracted from cells in both experimental and control groups utilizing TRIzol reagent (Invitrogen, Carlsbad, CA, United States). This RNA was then reverse-transcribed into complementary DNA using the PrimeScript RT reagent kit (Takara Bio, Inc., Shiga, Japan), following the manufacturer’s protocol. The expression levels of key genes related to bone and cementum formation were quantified via quantitative polymerase chain reaction (qPCR). The primer sequences for the differentiation markers used for the assay were as follows (5′–3′): bone sialoprotein (BSP; forward: TGCC TTGAGCCTGCTTCCT, reverse: CTGAGCAAAATTAA AGCAGTCTTCA), cementum attachment protein (CAP; forward: TTTTTCTGGTCGCGTGGACT, reverse: TCACCAGCAACTCCAACAGG), and cementum protein 1 (CEMP1; forward: GGGCACATCAAGCACTGACAG, reverse: CCCTTAGGAAGTGGCTGTCCAG). To ensure accurate quantification, the expression levels of these target genes were normalized against GAPDH, a housekeeping gene with the following sequence (5′-3′): (forward: TCAGCAATGCCTCCTGCAC, reverse: TCTGGG TGGCAGTGATGG); using the 2‑ΔΔCT method. For controls, untreated hPDLSCs were designated as the negative control, while cells incubated in StemMACS OsteoDiff Media (Miltenyi Biotec, Bergish Gladbach, Germany) -a standard osteogenic differentiation medium- served as the positive control. To enhance the reliability of the results, these experiments were conducted in triplicate.

### Evaluation of calcified nodule formation

The influence of bioceramic sealers on the ability of human periodontal ligament stem cells (hPDLSCs) to generate calcified nodules was investigated through Alizarin Red S staining. This assay replicated the cell seeding procedure and the experimental group design employed in the gene expression analyses, including both the negative control (cells grown in basal growth media) and the positive control (cells cultured in OsteoDiff media (Miltenyi Biotec)). Following a cultivation period of 21 days, the cells were fixed using 4% paraformaldehyde for 30 min. Subsequently, they were stained with Alizarin Red S (Sigma-Aldrich Corporation, St. Louis, MO, USA) to identify calcified nodules. The stained samples were then visually documented using an Olympus CKX41 light microscope (Olympus, Tokyo, Japan). Quantitative assessment of mineralization was conducted through colorimetric analysis, measuring the absorbance at 577 nm with a microplate reader. To ensure the reproducibility and validity of the findings, each treatment condition and time point was replicated in triplicate.

### Statistical methodology

Data were analyzed using GraphPad Prism software, version 8.1.0 (GraphPad Software Inc., San Diego, CA, USA). The results represent mean values derived from three independent experimental runs. Initially, the distribution of data was evaluated for normality using a Quantile-Quantile (Q-Q) plot. Based on the normality and homogeneity of variance assessments, statistical significance was determined either through one-way Analysis of Variance (ANOVA) with subsequent Tukey’s post-hoc test or via the Mann-Whitney U test. The threshold for statistical significance was set at a p-value of less than 0.05.

## Results

### Cell characterization and metabolic activity

**Fig. 1:**
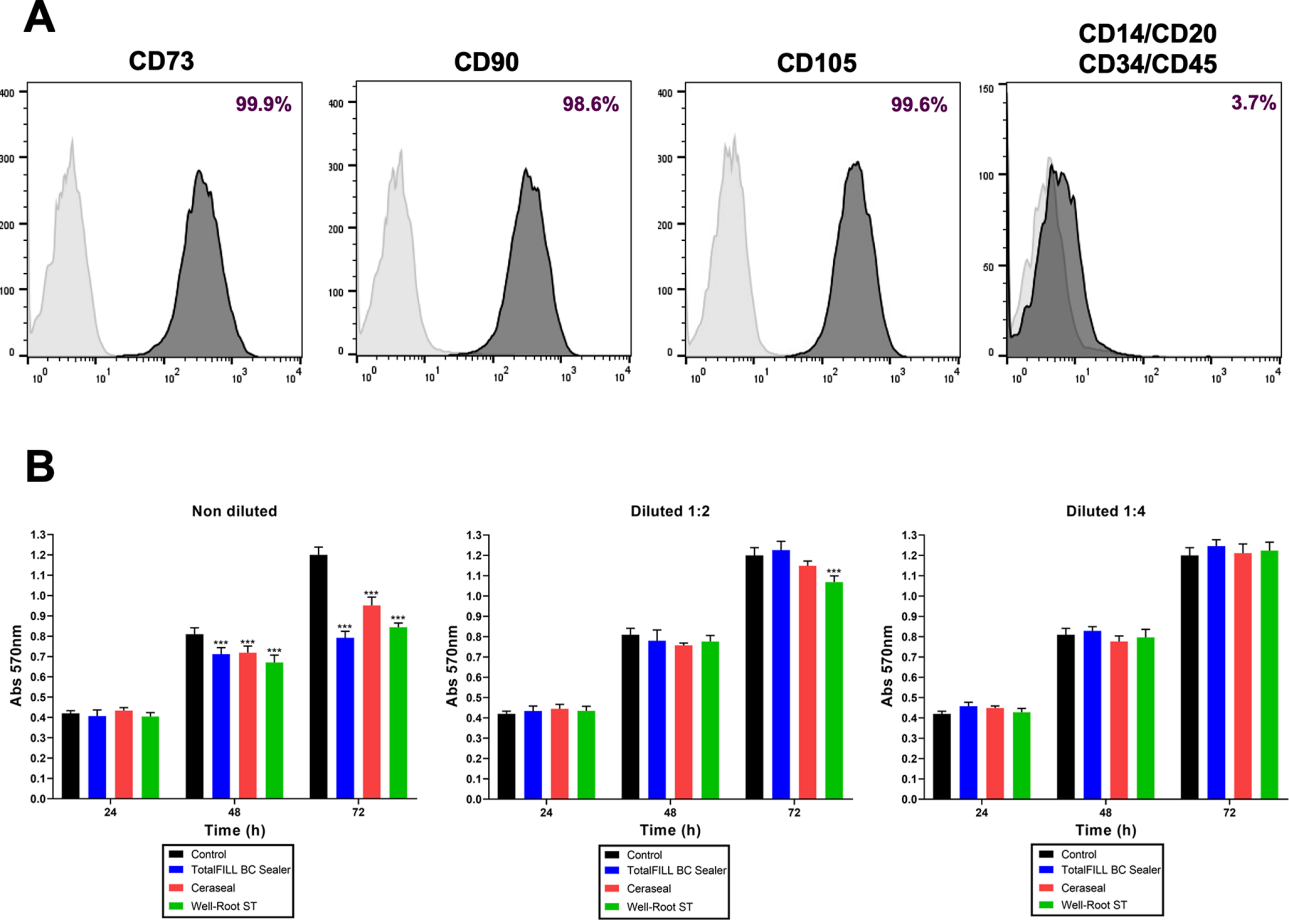
(**A**) Results from hPDLSC characterization. Marker expression is presented as a percentage (%). (**B**) Results from the cell metabollic assay (MTT) for the 1:1, 1:2 and 1:4 eluates of the tested sealers (TFbc, WR-ST, and CS) after 24, 48, and 72 h of culture with hPDLSCs. Data are presented absorbance values (570 nm) compared to the negative control group. *p < 0.05; **p < 0.01; ***p < 0.001

Human periodontal ligament stem cells (hPDLSCs) demonstrated evident expression of CD73, CD90, and CD105, while lacking expression of hematopoietic markers (CD14, CD20, CD34, CD45), thereby affirming their mesenchymal identity; as depicted in Fig. 1A. Metabolic activity assessment via the MTT assay (Fig. 1B) revealed that undiluted test materials exhibited significantly reduced metabolic activity compared to the control at both 48 and 72 h (*p* < 0.001). Conversely, dilutions at 1:2 and 1:4 ratios of the sealers showed no significant metabolic differences when compared to the control. However, a slight difference was noted with a 1:2 dilution of WR-ST at 72 h. Overall, bioceramic sealers exhibited a trend of increasing cell viability over time.

### Cell migration and cytoskeleton staining

**Fig. 2:**
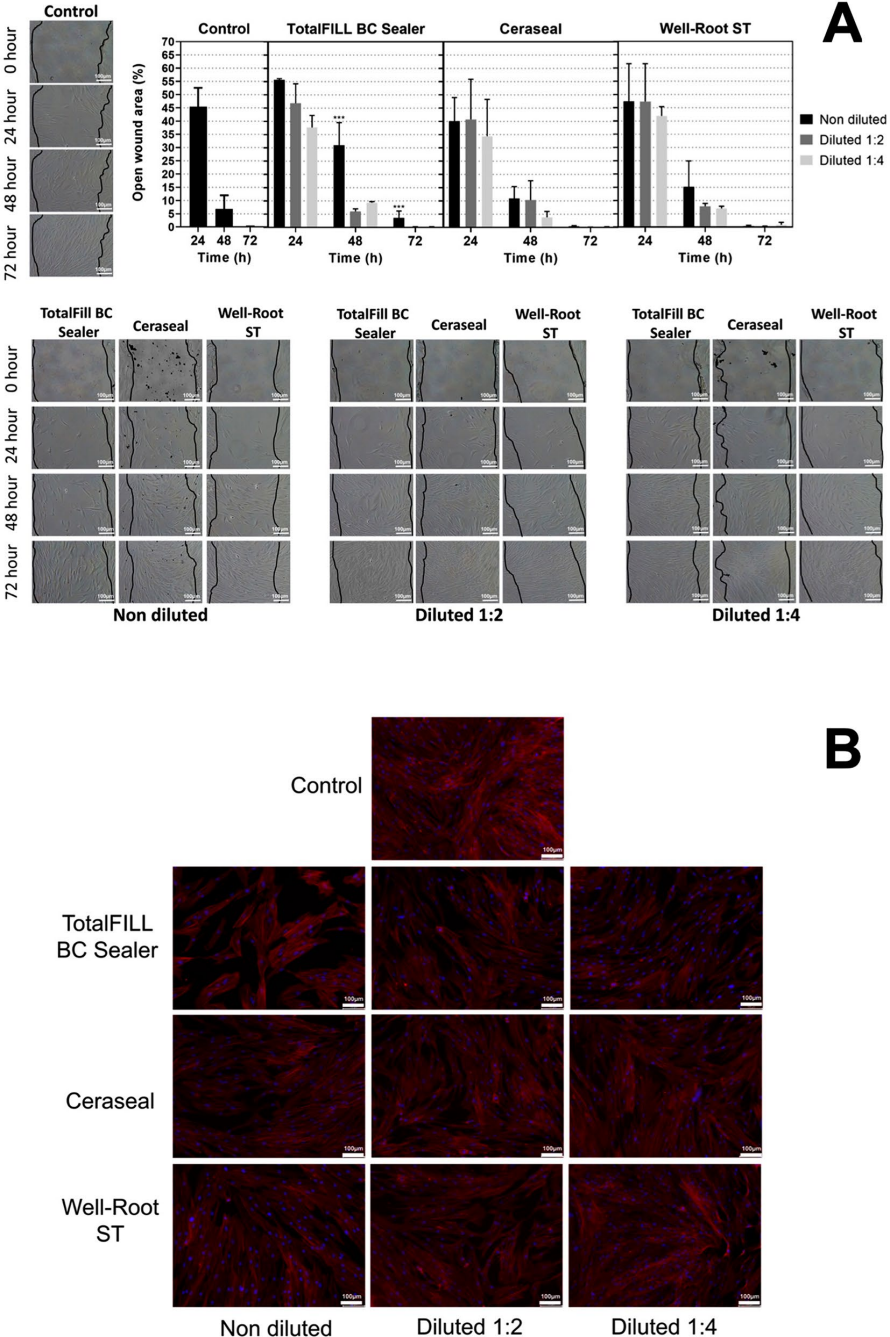
(**A**) Results from the cell migration assay (wound healing) for the 1:1, 1:2 and 1:4 eluates of the tested sealers (TFbc, WR-ST, and CS) after 24, 48, and 72 h of culture with hPDLSCs. Graphical results are presented as percentages of open wound areas compared to the negative control group. ***p < 0.001. (**B**) Results from the hPDLSC cytoskeleton staining (phaloidin-based assay) after 72 h of culture with 1:1 testes sealers (TFbc, WR-ST, and CS). Scale bar: 100 μm

Results from the wound healing assay indicate that CS and WR-ST did not markedly influence cell behavior at any observed time point relative to the control group. In contrast, cells exposed to undiluted TFbc displayed a slower wound closure rate at 48 h and 72 h compared to the control, with a significant difference noted at 48 h (*p* < 0.001), but not at 24 h. Overall, the materials under investigation facilitated wound closure, as illustrated in Fig. 2A and B. Phalloidin staining highlighted a significant cellular confluence accompanied by an upsurge in F-actin stress fibers and focal adhesion complexes across the samples treated with control, TFbc, CS, and WR-ST, as documented in Fig. 2C.

### SEM and Elisa assays

**Fig. 3:**
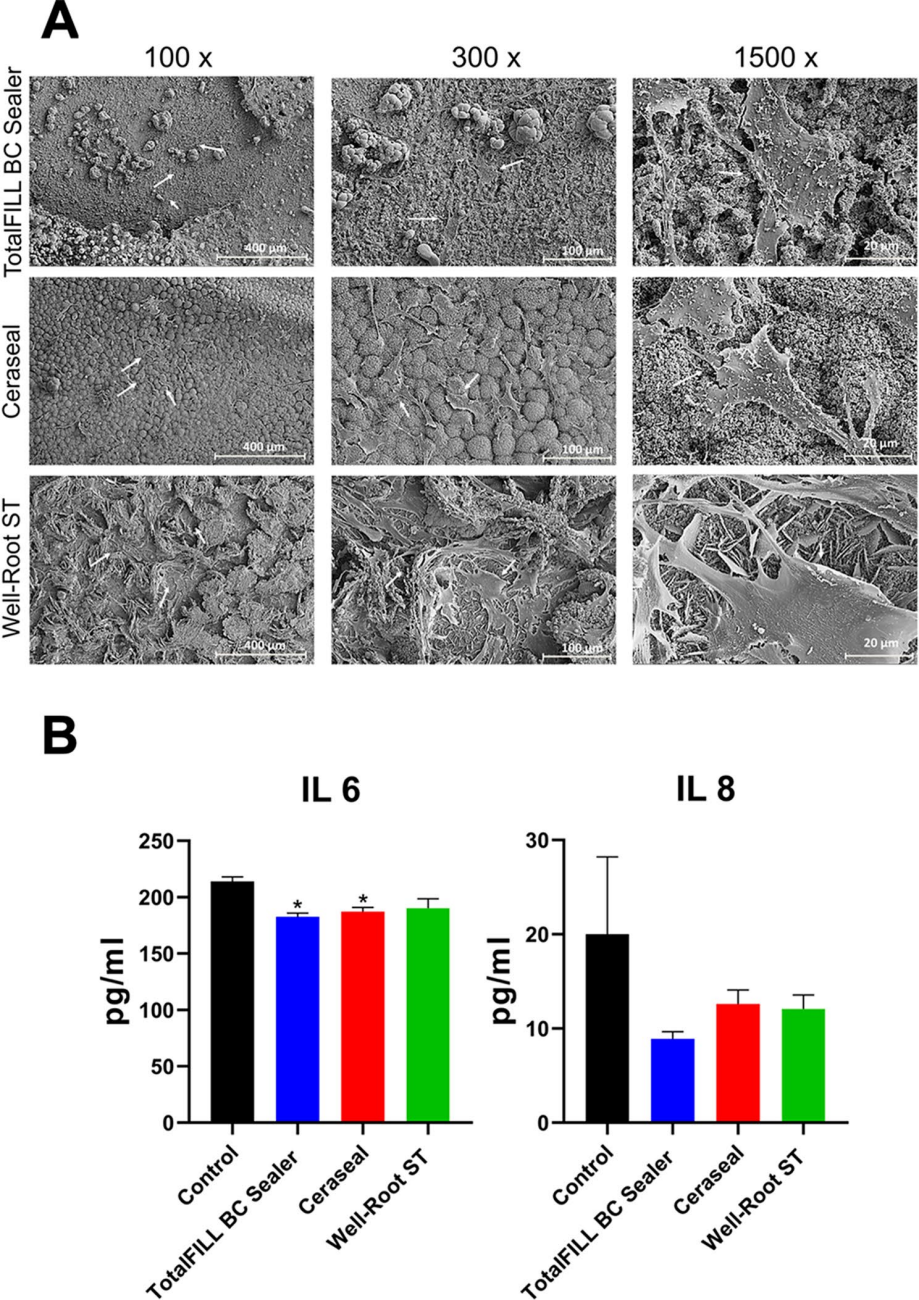
(**A**) SEM images (cell adhesion and morphology assay) after 72 h of culture of hPDLSCs seeded onto the surface of the tested sealer discs (TFbc, WR-ST, and CS). Magnifications: 100X, 300X, and 1500X. Scale bars: 400 μm, 100 μm, and 20 μm. (**B**) Results from the ELISA to assess hPDLSC expression of IL-6 and IL-8 after 72 h of culture with the testes sealers (TFbc, WR-ST, and CS) compared to the negative control. *p < 0.05; **p < 0.01; ***p < 0.001

Scanning electron microscopy (SEM) at 1500x magnification demonstrated that the experimental groups displayed cellular filopodia, facilitating attachment to the surface of the calcium silicate cement. Furthermore, these groups exhibited enhanced cell adhesion, evidenced by improved substrate attachment, cellular spreading, and cytoskeletal development within the niche-like structures of the cement (Fig. 3A). To assess the potential of bioceramic sealers to mitigate the synthesis of pro-inflammatory cytokines, the production levels of IL6 and IL8 were measured. Results indicated that IL6 levels were significantly reduced in the TFbc and CS groups compared to the control and WR-ST groups (*p* < 0.05) (Fig. 3B). However, there were no significant differences in IL8 production among all the groups tested (Fig. 3B).

### Gene expression and Alizarin Red staining

**Fig. 4:**
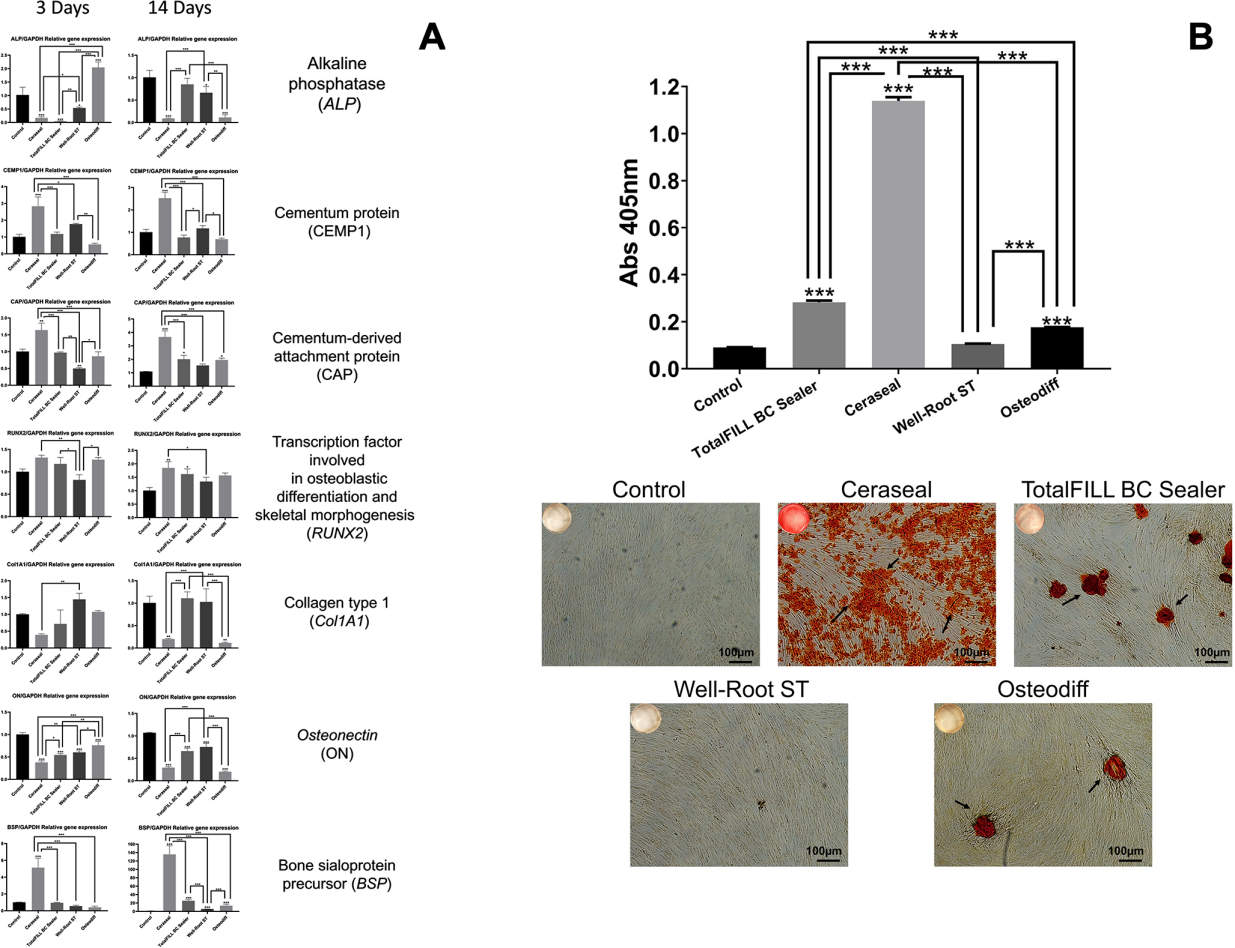
(**A**) RT-qPCR results for hPDLSCs marker expression after 3, 7, 14 and 21 days of culture with the tested sealers (TFbc, WR-ST, and CS), relative to the negative and positive control groups. *p < 0.05; **p < 0.01; ***p < 0.001. (**B**) Results from the Alizarin Red S staining assay of hPDLSCs after 21 days of culture with the tested sealers(TFbc, WR-ST, and CS), relative to the negative and positive control groups. *p < 0.05; **p < 0.01; ***p < 0.001. In both panels, asterisks above the bars indicate a significant difference with the negative control group; asterisks above the lines indicate a significant difference between the groups connected by the line

The impact of calcium silicate-based sealers on gene expression profiles related to osteogenic and cementogenic differentiation and mineralization was investigated using quantitative reverse transcription PCR (qRT-PCR) and Alizarin Red staining. Figure 4A reveals that on day 3, the expression levels of bone sialoprotein (BSP), cementum protein 1 (CEMP-1), calcium-binding protein (CAP), and runt-related transcription factor 2 (RUNX2) -a key transcription factor involved in osteoblastic differentiation and skeletal morphogenesis- were significantly elevated in the CS group compared to the control group (*p* < 0.01). By day 14, there was a marked upregulation of BSP, CEMP-1, and CAP in the CS group relative to the control (*p* < 0.01). The formation of mineralized nodules, indicative of osteogenic and cementogenic differentiation, was notably observed. After 21 days, Alizarin Red staining demonstrated significantly enhanced mineral deposition in the CS group compared to the TFbc, WR-ST, and Osteodiff groups (*p* < 0.001), as depicted in Fig. 4B. Collectively, these findings indicate that calcium silicate-based sealers, particularly CS and TFbc, are potent inducers of osteogenic and cementogenic differentiation in human periodontal ligament stem cells (hPDLSCs).

## Discussion

The current research investigated the biological and immunomodulatory impacts of three root canal sealers, revealing substantial variations. Consequently, this evidence led to the rejection of the null hypothesis. Specifically, the findings indicated that Ceraseal (CS) facilitated a notable decrease in the synthesis of pro-inflammatory cytokines such as IL-6 while concurrently promoting mineralization.

The assessment of material cytocompatibility through *in vitro evaluation* serves as a preliminary indicator of its suitability for clinical applications [16]. Accordingly, the biocompatibility of materials chosen for root canal treatment warrants careful consideration [17]. Such materials are expected to support the survival and proliferation of human periodontal ligament stem cells (hPDLSCs) within the periapical tissues, which possess intrinsic reparative capabilities. Recent advancements have focused on the development of calcium silicate-based cements and sealers, which are designed to exhibit bioactive properties [12, 13]. This is achieved by the release of calcium and hydroxide ions that contribute to the formation of hydroxyapatite, thereby facilitating periapical tissue repair [18]. Despite these developments, there remains a scarcity of data regarding the anti-inflammatory properties of these materials.

The ISO 10993-5 standard, as delineated by the International Organization for Standardization in 2009, establishes protocols for evaluating the cytotoxic effects of materials used in the medical field [9]. The current investigation adhered to the stipulations recommended by ISO 10993-5 designate the MTT assay as a viable method for assessing the cytotoxicity of dental materials. In addition, the application of the MTT reagent is a conventional approach in the cytotoxic evaluation of dental materials. Consistent with findings from previous research, our observations indicated that bioceramic sealers demonstrated a propensity for enhanced cell viability over time [18, 19].

Regarding cellular migration, literature suggests that the mechanisms underlying wound closure in the scratch assay encompass both cellular proliferation and migration capabilities [20]. Indeed, the interaction between biomaterials and cellular structures can precipitate cellular degeneration and impede the process of wound healing [21]. In the context of this study, cells treated with bioceramics exhibited responses analogous to those observed in the untreated control group throughout the duration of the wound healing assay [20, 22]. Furthermore, an initial assessment of cytotoxicity was conducted through a morphological analysis utilizing immunofluorescence techniques. According to Rohr et al. [23], cytotoxic constituents present in dental materials are known to cause disruptions in cell integrity, the presence of pyknotic nuclei, and alterations in cytoskeleton organization. Despite these potential adverse effects, cytological alterations indicative of cytotoxic responses, particularly those concerning the cytoskeleton, were not observed in the materials tested in this study. This absence of cytological damage is consistent with the results obtained from both the MTT assay and the migration assays, reinforcing the outcome that the tested materials are biocompatible under the conditions examined.

In accordance with the aforementioned findings, our SEM results unveiled the presence of cellular filopodia, which facilitate the anchoring of cells to the surface of the materials. The role of calcium in this biological interaction is crucial, particularly in the context of fibroblast adhesion [24, 25]. Enhanced cellular attachment has been correlated with the release of calcium ions (Ca^2+^) from endodontic sealers. As documented in Table 1, the inclusion of Ca^2+^ within the composition of these materials likely accounts for the observed increase in cell attachment. This correlation underscores the significance of calcium as a pivotal element in promoting adhesion at the cellular level [26], thereby supporting the biocompatibility and therapeutic efficacy of the materials used.

The anti-inflammatory response plays a pivotal role in tissue repair, serving as a fundamental indicator of the immune system’s ability to eliminate cellular debris and enhance cellular differentiation [27]. Bioceramic sealers, due to their high calcium ion (Ca^2+^) content, may alkalize the culture medium. Such an alkaline environment is hypothesized to prompt human periodontal ligament stem cells (hPDLSCs) to reduce the synthesis of pro-inflammatory cytokines. Our findings indicate that CS and TFBc substantially diminish the production of IL-6 and interleukin-8 (IL-8). IL-6 plays an integral role in bone remodeling, as well as in the activation and differentiation of immune cells and osteoclasts. Previous research has established a link between elevated IL-6 levels and the occurrence of symptomatic and active endodontic lesions [28]. Moreover, IL-8 has been shown to promote the inflammatory response and the progression of such lesions. The interplay of these cytokines, particularly IL-6 and IL-8, is likely to significantly impact the healing processes of endodontic lesions by modulating the immune response and facilitating tissue remodeling [29].

In the context of the RT-qPCR assay, a consistent pattern was observed. Following established methodologies from previous studies, several genes were utilized as markers for osteogenic and cementogenic differentiation [18, 30–32]. CS were able to induce overexpression of the BSP, CEMP-1, and CAP. These markers are involved in early differentiation into osteoblasts/cementoblasts (3–14 days) [30]. Bone sialoprotein (BSP) is a well-recognized marker that plays a critical role in identifying osteogenic differentiation. Conversely, cementum protein 1 (CEMP1) serves as a specific marker for identifying cementoblasts and their progenitors [33]. In terms of periapical regeneration, cementum production is essential for the formation of replacement tissue, often termed the “biological seal.” This biological seal is regarded as the optimal environment for endodontic treatment repair, as emphasized by Sanz et al. [34]. Thus, the neoformation of cementum facilitated by endodontic materials is deemed the optimal mechanism for achieving successful periapical regeneration. This process is vital for ensuring the restoration and maintenance of periapical tissue health, thereby contributing to the overall effectiveness of endodontic treatments.

Mineralization of the extracellular matrix is a critical indicator of bone regeneration in vitro. In this study, hPDLSCs treated with CS exhibited a greater intensity of Alizarin Red staining, indicative of increased mineralization nodule formation, compared to untreated cells or those exposed to TFbc, WR-ST, and Osteodiff over the same period. Previous studies have demonstrated that enhanced nucleation activity and elevated calcium release promote mineralization [24]. Furthermore, inorganic ions released by bioceramic materials serve as vital signaling molecules and regulatory factors involved in various cellular activities which are essential for bone homeostasis [32]. These elements likely contributed to the pronounced bone differentiation observed with CS and TFbc treatments, aligning with previous findings on the osteogenic properties of bioceramic root canal sealers [18, 19].

However, this study has some limitations. Although these results cannot be directly applied to clinical situations in humans, they are scientifically significant because they represent an appropriate prototype for evaluating various initial features of dental materials, as observed in previous similar in vitro studies [35]. As a result, further research using in vivo animal models is necessary to confirm the results [36, 37].

## Conclusions

All the tested sealers exhibited an adequate cytocompatibility. CS significantly enhances cell differentiation by upregulating the expression of key genes associated with bone and cementum formation. Additionally, CS was observed to facilitate the mineralization of the extracellular matrix effectively. In contrast, the effects of TFbc and WR-ST on these processes were less pronounced compared to CS. Furthermore, both CS and TFbc exhibited an anti-inflammatory potential, contributing to their potential therapeutic benefits in regenerative endodontics.

## Data Availability

No datasets were generated or analysed during the current study.
